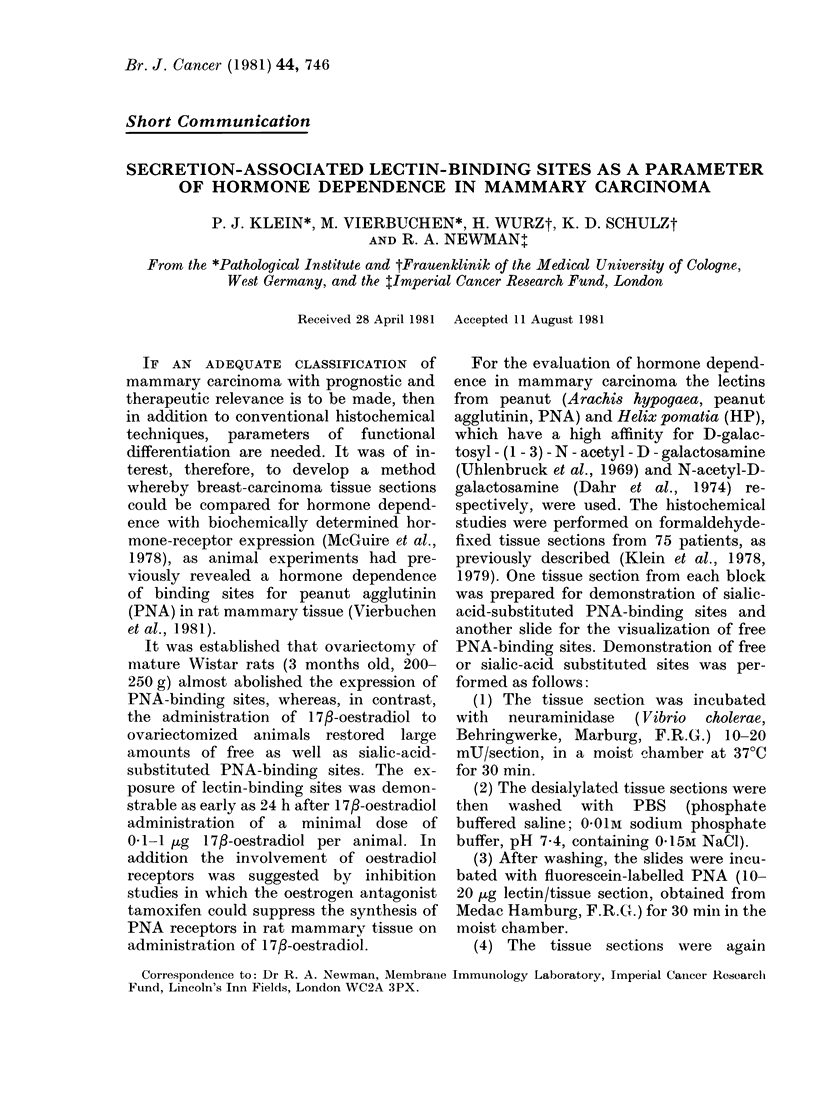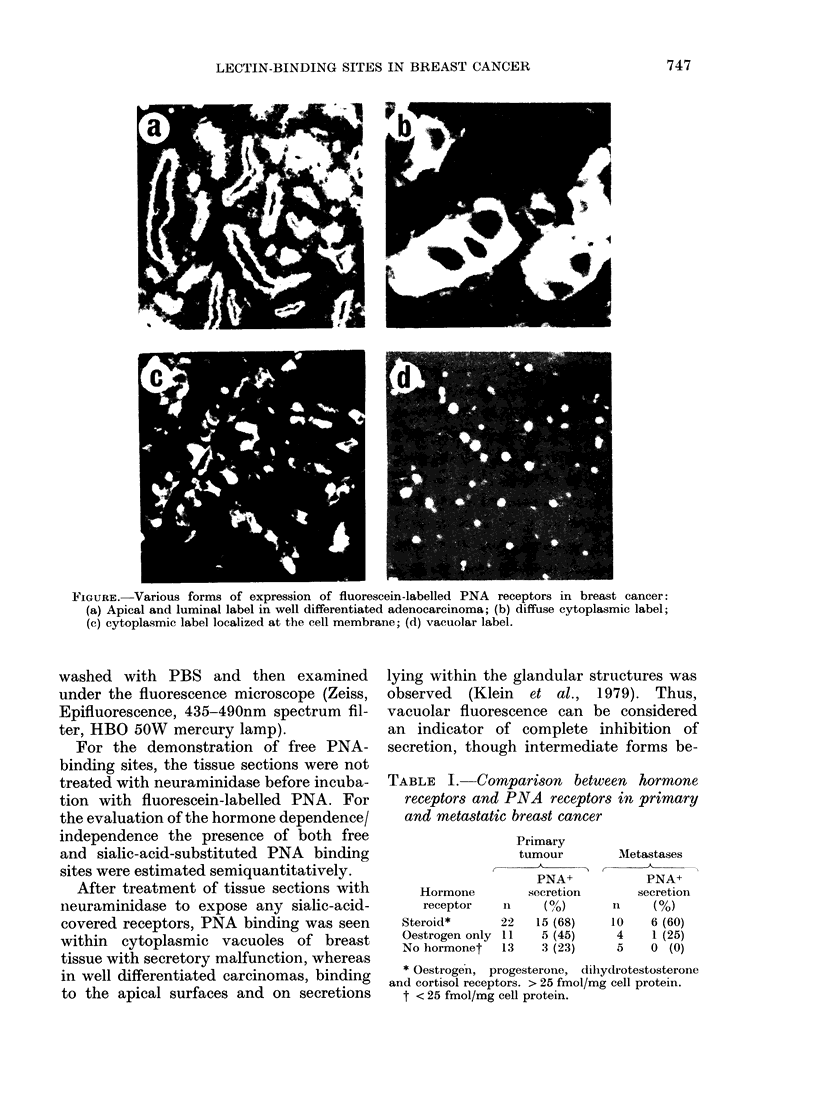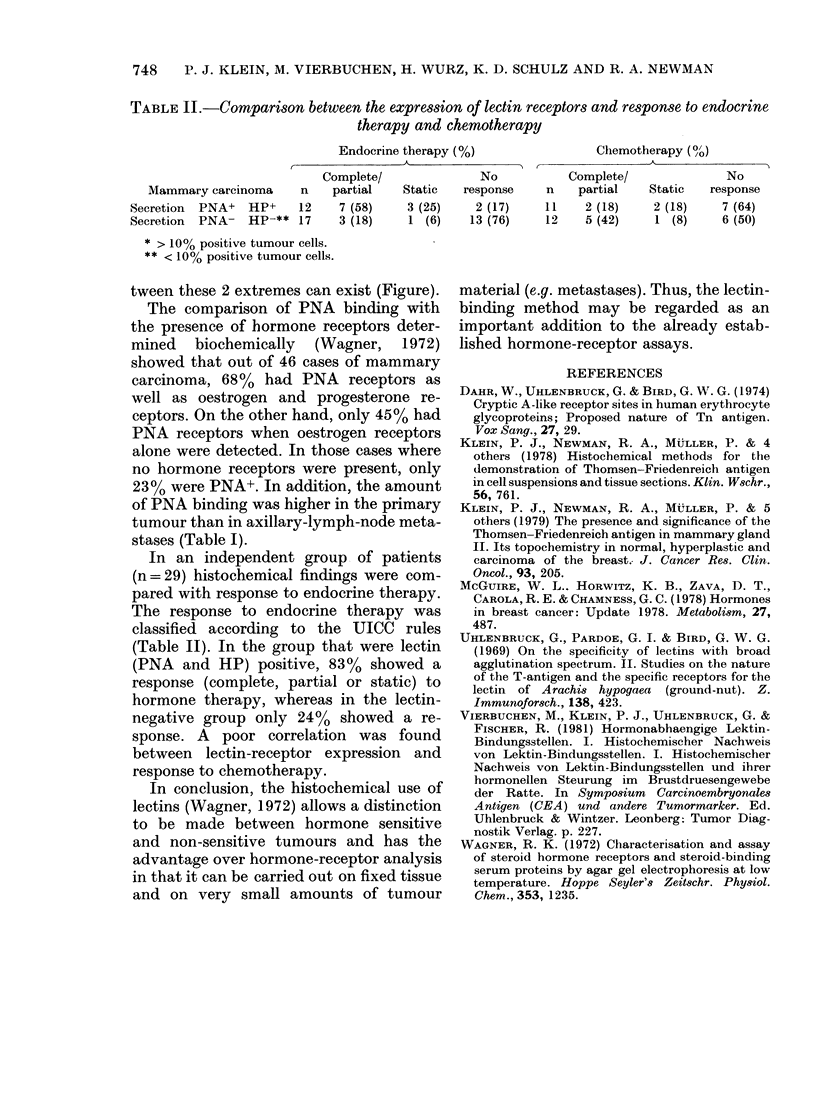# Secretion-associated lectin-binding sites as a parameter of hormone dependence in mammary carcinoma.

**DOI:** 10.1038/bjc.1981.262

**Published:** 1981-11

**Authors:** P. J. Klein, M. Vierbuchen, H. Wurz, K. D. Schulz, R. A. Newman

## Abstract

**Images:**


*Br. J. Cancer* (1981) **44**, 746

***Short Communication***

### SECRETION-ASSOCIATED LECTIN-BINDING SITES AS A PARAMETER OF HORMONE DEPENDENCE IN MAMMARY CARCINOMA

P. J. KLEIN*, M. VIERBUCHEN*, H. WURZ†, K. D. SCHULZ† AND R. A. NEWMAN‡

*From the *Pathological Institute and †Frauenklinik of the Medical University of Cologne, West Germany, and the ‡Imperial Cancer Research Fund, London*

Received 28 April 1981 Accepted 11 August 1981

IF AN ADEQUATE CLASSIFICATION of mammary carcinoma with prognostic and therapeutic relevance is to be made, then in addition to conventional histochemical techniques, parameters of functional differentiation are needed. It was of interest, therefore, to develop a method whereby breast-carcinoma tissue sections could be compared for hormone dependence with biochemically determined hormone-receptor expression (McGuire *et al.*, 1978), as animal experiments had previously revealed a hormone dependence of binding sites for peanut agglutinin (PNA) in rat mammary tissue (Vierbuchen *et al.*, 1981).

It was established that ovariectomy of mature Wistar rats (3 months old, 200–250 g) almost abolished the expression of PNA-binding sites, whereas, in contrast, the administration of 17β-oestradiol to ovariectomized animals restored large amounts of free as well as sialic-acid-substituted PNA-binding sites. The exposure of lectin-binding sites was demonstrable as early as 24 h after 17β-oestradiol administration of a minimal dose of 0·1–1 μg 17β-oestradiol per animal. In addition the involvement of oestradiol receptors was suggested by inhibition studies in which the oestrogen antagonist tamoxifen could suppress the synthesis of PNA receptors in rat mammary tissue on administration of 17β-oestradiol.

For the evaluation of hormone dependence in mammary carcinoma the lectins from peanut (*Arachis hypogaea*, peanut agglutinin, PNA) and *Helix pomatia* (HP), which have a high affinity for D-galactosyl-(1-3)-N-acetyl-D-galactosamine (Uhlenbruck *et al.*, 1969) and N-acetyl-D-galactosamine (Dahr *et al.*, 1974) respectively, were used. The histochemical studies were performed on formaldehyde-fixed tissue sections from 75 patients, as previously described (Klein *et al.*, 1978, 1979). One tissue section from each block was prepared for demonstration of sialic-acid-substituted PNA-binding sites and another slide for the visualization of free PNA-binding sites. Demonstration of free or sialic-acid substituted sites was performed as follows:

(1) The tissue section was incubated with neuraminidase (*Vibrio cholerae*, Behringwerke, Marburg, F.R.G.) 10–20 mU/section, in a moist chamber at 37°C for 30 min.

(2) The desialylated tissue sections were then washed with PBS (phosphate buffered saline; 0·01M sodium phosphate buffer, pH 7·4, containing 0·15M NaCl).

(3) After washing, the slides were incubated with fluorescein-labelled PNA (10–20 μg lectin/tissue section, obtained from Medac Hamburg, F.R.G.) for 30 min in the moist chamber.

(4) The tissue sections were again

Correspondence to: Dr R. A. Newman, Membrane Immunology Laboratory, Imperial Cancer Research Fund, Lincoln's Inn Fields, London WC2A 3PX.

FIGURE.—Various forms of expression of fluorescein-labelled PNA receptors in breast cancer: (a) Apical and luminal label in well differentiated adenocarcinoma; (b) diffuse cytoplasmic label; (c) cytoplasmic label localized at the cell membrane; (d) vacuolar label.

washed with PBS and then examined under the fluorescence microscope (Zeiss, Epifluorescence, 435–490nm spectrum filter, HBO 50W mercury lamp).

For the demonstration of free PNA-binding sites, the tissue sections were not treated with neuraminidase before incubation with fluorescein-labelled PNA. For the evaluation of the hormone dependence/independence the presence of both free and sialic-acid-substituted PNA binding sites were estimated semiquantitatively.

After treatment of tissue sections with neuraminidase to expose any sialic-acid-covered receptors, PNA binding was seen within cytoplasmic vacuoles of breast tissue with secretory malfunction, whereas in well differentiated carcinomas, binding to the apical surfaces and on secretions lying within the glandular structures was observed (Klein *et al.*, 1979). Thus, vacuolar fluorescence can be considered an indicator of complete inhibition of secretion, though intermediate forms be-

TABLE I.—*Comparison between hormone receptors and PNA receptors in primary and metastatic breast cancer*

| | Primary tumour | | Metastases | |
|---|---|---|---|---|
| Hormone receptor | n | $PNA^+$ secretion (%) | n | $PNA^+$ secretion (%) |
| Steroid* | 22 | 15 (68) | 10 | 6 (60) |
| Oestrogen only | 11 | 5 (45) | 4 | 1 (25) |
| No hormone† | 13 | 3 (23) | 5 | 0 (0) |

* Oestrogen, progesterone, dihydrotestosterone and cortisol receptors. > 25 fmol/mg cell protein.
† < 25 fmol/mg cell protein.

TABLE II.—*Comparison between the expression of lectin receptors and response to endocrine therapy and chemotherapy*

| Mammary carcinoma | Endocrine therapy (%) | | | | Chemotherapy (%) | | | |
|---|---|---|---|---|---|---|---|---|
| | n | Complete/ partial | Static | No response | n | Complete/ partial | Static | No response |
| Secretion PNA+ HP+ | 12 | 7 (58) | 3 (25) | 2 (17) | 11 | 2 (18) | 2 (18) | 7 (64) |
| Secretion PNA− HP−** | 17 | 3 (18) | 1 (6) | 13 (76) | 12 | 5 (42) | 1 (8) | 6 (50) |

* > 10% positive tumour cells.
** < 10% positive tumour cells.

tween these 2 extremes can exist (Figure).

The comparison of PNA binding with the presence of hormone receptors determined biochemically (Wagner, 1972) showed that out of 46 cases of mammary carcinoma, 68% had PNA receptors as well as oestrogen and progesterone receptors. On the other hand, only 45% had PNA receptors when oestrogen receptors alone were detected. In those cases where no hormone receptors were present, only 23% were PNA+. In addition, the amount of PNA binding was higher in the primary tumour than in axillary-lymph-node metastases (Table I).

In an independent group of patients (n = 29) histochemical findings were compared with response to endocrine therapy. The response to endocrine therapy was classified according to the UICC rules (Table II). In the group that were lectin (PNA and HP) positive, 83% showed a response (complete, partial or static) to hormone therapy, whereas in the lectin-negative group only 24% showed a response. A poor correlation was found between lectin-receptor expression and response to chemotherapy.

In conclusion, the histochemical use of lectins (Wagner, 1972) allows a distinction to be made between hormone sensitive and non-sensitive tumours and has the advantage over hormone-receptor analysis in that it can be carried out on fixed tissue and on very small amounts of tumour material (*e.g.* metastases). Thus, the lectin-binding method may be regarded as an important addition to the already established hormone-receptor assays.

### REFERENCES

DAHR, W., UHLENBRUCK, G. & BIRD, G. W. G. (1974) Cryptic A-like receptor sites in human erythrocyte glycoproteins; Proposed nature of Tn antigen. *Vox Sang.*, **27**, 29.

KLEIN, P. J., NEWMAN, R. A., MÜLLER, P. & 4 others (1978) Histochemical methods for the demonstration of Thomsen–Friedenreich antigen in cell suspensions and tissue sections. *Klin. Wschr.*, **56**, 761.

KLEIN, P. J., NEWMAN, R. A., MÜLLER, P. & 5 others (1979) The presence and significance of the Thomsen–Friedenreich antigen in mammary gland II. Its topochemistry in normal, hyperplastic and carcinoma of the breast. *J. Cancer Res. Clin. Oncol.*, **93**, 205.

McGUIRE, W. L., HORWITZ, K. B., ZAVA, D. T., CAROLA, R. E. & CHAMNESS, G. C. (1978) Hormones in breast cancer: Update 1978. *Metabolism*, **27**, 487.

UHLENBRUCK, G., PARDOE, G. I. & BIRD, G. W. G. (1969) On the specificity of lectins with broad agglutination spectrum. II. Studies on the nature of the T-antigen and the specific receptors for the lectin of *Arachis hypogaea* (ground-nut). *Z. Immunoforsch.*, **138**, 423.

VIERBUCHEN, M., KLEIN, P. J., UHLENBRUCK, G. & FISCHER, R. (1981) Hormonabhaengige Lektin-Bindungsstellen. I. Histochemischer Nachweis von Lektin-Bindungsstellen. I. Histochemischer Nachweis von Lektin-Bindungsstellen und ihrer hormonellen Steurung im Brustdruesengewebe der Ratte. In *Symposium Carcinoembryonales Antigen (CEA) und andere Tumormarker*. Ed. Uhlenbruck & Wintzer. Leonberg: Tumor Diagnostik Verlag. p. 227.

WAGNER, R. K. (1972) Characterisation and assay of steroid hormone receptors and steroid-binding serum proteins by agar gel electrophoresis at low temperature. *Hoppe Seyler's Zeitschr. Physiol. Chem.*, **353**, 1235.